# Rapid Identification of Dendrobium Species Using Near-Infrared Hyperspectral Imaging Technology

**DOI:** 10.3390/s25185625

**Published:** 2025-09-09

**Authors:** Kaixuan Li, Yijun Guo, Haosheng Zhong, Yiqi Jin, Bin Li, Huimin Fang, Lijian Yao, Chao Zhao

**Affiliations:** 1 College of Optical, Mechanical and Electrical Engineering, Zhejiang A&F University, Hangzhou 311300, China; 2023612021010@stu.zafu.edu.cn (K.L.); pop15779972603@outlook.com (Y.G.); 20130083@zafu.edu.cn (B.L.); 2 Zhoushan Special Equipment Inspection Research Institute, Zhoushan 316021, China; 13656800858@163.com; 3 Jixian Honors College, Zhejiang A&F University, Hangzhou 311300, China; a779733711@163.com; 4 School of Agricultural Engineering, Jiangsu University, Zhenjiang 212013, China; fanghuimin@ujs.edu.cn

**Keywords:** hyperspectral imaging technology, SVM, *Dendrobium officinale*, CARS, SPA

## Abstract

*Dendrobium officinale* is a valuable Chinese medicinal herb, but distinguishing it from other Dendrobium species after processing is challenging, leading to low classification accuracy and time-consuming analysis. This study proposes a rapid classification model based on near-infrared hyperspectral imaging (NIR-HSI), incorporating data preprocessing and feature wavelength selection. Five Dendrobium species—*D. officinale*, *D. aphyllum*, *D. chrysanthum*, *D. fimbriatum*, and *D. thyrsiflorum*—were used. Spectral preprocessing techniques like normalization and smoothing were applied, and Support Vector Machine (SVM) models were constructed. Normalization improved both accuracy and stability, with the full-spectrum Normalize-SVM model achieving 97% accuracy for calibration and 88% for prediction. *D. chrysotoxum* performed best, with all metrics reaching 100%, while *D. aphyllum* had poor classification (40% recall and 51.74% F1 score). To improve efficiency and performance, feature wavelength selection was performed using Competitive Adaptive Reweighted Sampling (CARS) and Successive Projections Algorithm (SPA). The CARS-Normalize-SVM model yielded the best results: 98% accuracy for calibration and 96% for prediction, improving by 1% and 8%, respectively. D. aphyllum’s classification also improved significantly, with a 100% recall rate and 95.24% F1 score. These findings highlight hyperspectral imaging’s potential for rapid Dendrobium species identification, supporting future quality control and market supervision.

## 1. Introduction

*Dendrobium officinale*, a highly valued traditional Chinese medicinal herb, is mainly cultivated in Zhejiang, Anhui, and Yunnan provinces. It is rich in polysaccharides, alkaloids, and flavonoids, benefiting the lungs, digestion, fluid production, and immune regulation. Due to rising demand, fraudulent practices have emerged, with manufacturers using non-*D. officinale* species in supplements [1,2]. In 2024, investigations revealed common Dendrobium species processed into granules falsely labeled as *D. officinale* products [3]. Strict quality standards and reliable identification methods are needed to combat adulteration and protect consumers [4,5]. Thus, there is an urgent need for accurate methods to identify Dendrobium species to prevent fraudulent practices.

Common methods for identifying *D. officinale* include microscopic identification, chemical fingerprinting, DNA molecular techniques, and various spectroscopic methods such as NIR, FTIR, and Raman spectroscopy [6,7,8]. Microscopic identification relies on anatomical features but is subjective and requires expertise. DNA methods like RAPD and ISSR can differentiate species but are time-consuming and not suited for rapid identification [9,10]. Chemical fingerprinting, including HPLC, can differentiate *D. officinale* from counterfeits based on key bioactive compounds [11], but it is destructive and requires complex sample preparation [12]. These traditional identification methods have limitations in terms of speed, accuracy, or destructiveness.

NIR spectroscopy provides a rapid, non-destructive solution, and studies show its effectiveness in distinguishing *D. officinale* from similar species [13,14]. However, spectral data alone can limit prediction accuracy. FTIR analyzes chemical components like polysaccharides and flavonoids, aiding species differentiation, with high accuracy achieved through PLS-DA [15]. However, FTIR’s sensitivity is reduced in complex samples. Raman spectroscopy, although useful for detecting molecular vibrations, has limited effectiveness with closely related species due to overlapping spectral signals [16,17,18]. While NIR, FTIR, and Raman offer useful methods, their limitations hinder accurate identification in complex samples.

NIR hyperspectral imaging (NIR-HSI) combines spectral and spatial data, improving identification accuracy. Studies have shown that NIR-HSI can classify *D. officinale* with high accuracy, making it a promising tool for traceability and authenticity verification [19]. NIR-HSI is a promising, accurate, and non-destructive method for identifying *D. officinale*.

Near-infrared hyperspectral imaging (NIR-HSI) offers significant advantages in detecting *Dendrobium officinale*, gaining attention in both research and practical applications. Compared to traditional techniques like gas chromatography, mass spectrometry, or standalone near-infrared spectroscopy, which require complex, time-consuming, and often destructive sample preparation, NIR-HSI is faster, high-throughput, and non-destructive. It combines the chemical profiling of near-infrared spectroscopy with high-resolution imaging, enabling precise identification and quantitative analysis of morphological features, species differentiation, geographical origin, and key bioactive components such as polysaccharides, alkaloids, and amino acids [20,21,22]. NIR-HSI allows rapid data acquisition, scanning and analyzing large sample sets in minutes, improving efficiency for applications like origin tracing, quality grading, and adulteration detection [23,24,25]. When paired with machine learning and chemometric algorithms (e.g., PCA, PLSR, SVM), it generates highly accurate predictive models for automated analysis [26]. Thus, NIR-HSI is highly effective in distinguishing different Dendrobium species.

Various methods are used for identifying *Dendrobium officinale*, including microscopic identification, chemical fingerprinting (HPLC, GC), DNA molecular identification, and spectroscopic techniques such as NIR, FTIR, Raman, and hyperspectral imaging (HSI) [27]. Microscopic identification requires expertise, DNA methods are time-consuming, and chemical fingerprinting is destructive and complex. While NIR and FTIR are non-destructive and rapid, they face challenges with sensitivity and complex samples, and Raman spectroscopy struggles with closely related species. In contrast, NIR-HSI combines spectral and spatial data for faster, non-destructive, and highly accurate identification, making it ideal for species differentiation, quality control, and adulteration detection [28]. This study addresses the limitations of current methods by using NIR-HSI for rapid, non-destructive discrimination of Dendrobium species. By analyzing spectral characteristics and applying preprocessing and feature wavelength selection, a robust classification model will be developed to authenticate *D. officinale* and detect adulterants, supporting quality control standards in the health food industry.

## 2. Materials and Methods

### 2.1. Sample Collection

#### 2.1.1. Collection of Dendrobium Samples

A total of 200 samples were used in this study, comprising five Dendrobium species: *Dendrobium officinale, Dendrobium aphyllum*, *Dendrobium chrysanthum*, *Dendrobium fimbriatum*, and *Dendrobium thyrsiflorum*, with 40 samples collected for each species. Among the 200 samples used in this study, 30 samples of each of the five Dendrobium species, totaling 150 samples, were collected for spectral information in December 2024. Their spectral data was used to allocate the calibration set and prediction set for this study. In August 2025, spectral information of 50 samples from 10 different types of dendrobium was collected as an independent validation set. The spectral data of the same type of dendrobium was purchased from the same source, and the experimental procedures required were the same. Samples of *D. officinale* were obtained from Lingxuan Agricultural Development Co., Ltd., Yueqing, Zhejiang Province, China. *D. fimbriatum* samples were sourced from Kangmei Pharmaceutical Co., Ltd., Puning, Guangdong Province, China. *D. chrysanthum* samples were collected from Kangyiyin Biotechnology Co., Ltd., Bozhou, Anhui Province, China. Samples of *D. thyrsiflorum* and *D. aphyllum* were purchased from Xiaoliu Orchid Trading Department, Wenjiang District, Chengdu, Sichuan Province, China.

#### 2.1.2. Preparation of Dendrobium Samples

After sample collection, fresh Dendrobium stems free from pests and diseases were selected and thoroughly cleaned to remove surface soil and impurities. The leaves and roots of the cleaned samples from the five Dendrobium species were removed, retaining only the stems. These were then dried in a constant-temperature oven at 70 °C. The sample weight was measured every hour. Once the weight change between two consecutive measurements became minimal, the drying status was further verified according to the atmospheric pressure drying method described in GB/T 5009.3-2016, “Determination of Moisture in Food” [29]. Specifically, 5 g of the sample was ground into particles with diameters and lengths no greater than 3 mm and evenly spread in a flat-bottom weighing bottle that had been pre-dried to a constant weight. The sample layer thickness did not exceed 5 mm (10 mm for loosely packed samples). The sample was precisely weighed, then dried with the bottle cap open at 100–105 °C for 5 h. After cooling in a desiccator for 30 min, it was weighed again. The drying and weighing cycle was repeated until the difference between two successive weights was less than 5 mg. In this study, the Dendrobium stems were dried at 70 °C for 12 h. After grinding into fine particles, the moisture content was evaluated following the GB/T 5009.3-2016 protocol. The weight difference between two consecutive measurements was 4.3 mg, confirming that the samples were sufficiently dried [30].

**Figure 1:**
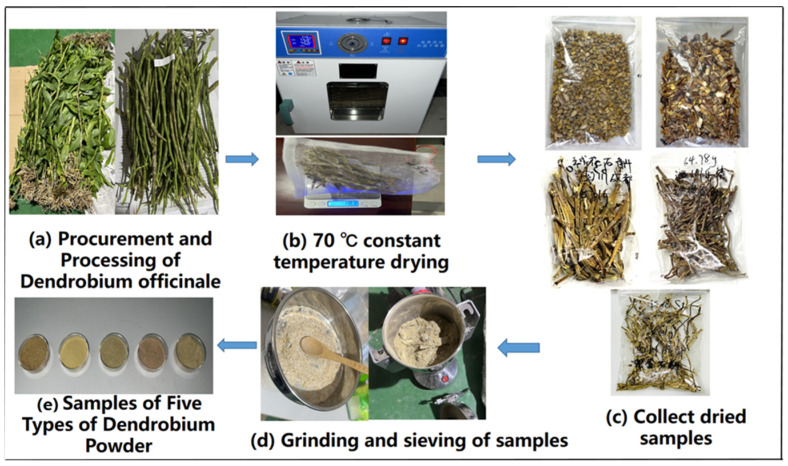
Preparation process of Dendrobium samples.

The dried Dendrobium stems were ground into powder using a grinder. The resulting powder was sieved through an 80-mesh screen, and the fine particles from each species were individually sealed, packaged, and stored under refrigeration for further use. The sample preparation procedure is illustrated in Figure 1. In this study, abbreviations based on the Latin names of the Dendrobium species are used to represent the five types: DA for *D. aphyllum*, DF for *D. fimbriatum*, DT for *D. thyrsiflorum*, DC for *D. chrysanthum*, and DO for *D. officinale*.

### 2.2. Near-Infrared Hyperspectral Imaging System

#### 2.2.1. Components of Near-Infrared Hyperspectral System

**Figure 2:**
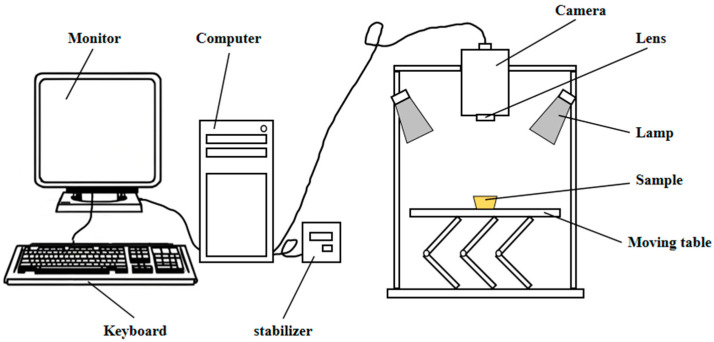
Hyperspectral imaging system.

The near-infrared hyperspectral imaging (NIR-HSI) system used in this study is shown in Figure 2. The system primarily consists of a motorized translation stage, halogen-tungsten light sources, a hyperspectral camera, a lens, a stabilizer, a computer, and a monitor. The technical specifications of the system components are listed in Table 1.

#### 2.2.2. The Advantages of Near-Infrared Hyperspectral Imaging Compared to RGB Imaging

Traditional RGB imaging can only capture color and morphological differences in samples within the visible light range, and can only serve as preliminary appearance characterization, unable to reveal changes in their internal chemical composition; In contrast, near-infrared hyperspectral imaging (NIR-HS) can record characteristic absorption peaks of chemical bonds such as O-H, N-H, C-H, etc., providing a spectral “fingerprint” directly related to functional components such as water, polysaccharides, alkaloids, etc., which theoretically has significant advantages. Taking *Dendrobium officinale* as an example, different varieties often exhibit similar appearance features in RGB images, making it difficult to distinguish their intrinsic quality. However, NIR-HSI images can clearly display differences in moisture content at 1450 nm and absorption characteristics of secondary metabolites near 2100 nm after spectral analysis, forming a chemical composition distribution map in experimental comparison, intuitively revealing the invisible intrinsic differences in RGB, thereby significantly enhancing the reliability of quality evaluation and theoretical research.

### 2.3. Hyperspectral Data Acquisition of Dendrobium Samples

Prior to spectral data acquisition, the light source was turned on 30 min in advance to allow for preheating and stabilization, minimizing the influence of environmental factors. During hyperspectral scanning, factors such as exposure time, light intensity, and the surface texture, shape, and color of the Dendrobium powder samples can affect the spatial and dimensional resolution of the captured images. Therefore, it was necessary to adjust the focus and configure appropriate scanning parameters before image acquisition. After repeated focus adjustments, the final imaging parameters were set as follows: object distance of 400 mm, light source current of 5.0 A, scanning speed of 150 μm/s, scan line length of 70 mm, and exposure time of 25 ms. The resulting hyperspectral images had a spectral resolution of 300 pixels × 310 pixels × 248 bands. During the experiment, six Dendrobium powder samples were placed sequentially on the sample stage for each group, and hyperspectral images were captured one by one. Hyperspectral data acquisition was performed using the imaging system shown in Figure 2.

**Figure 3:**
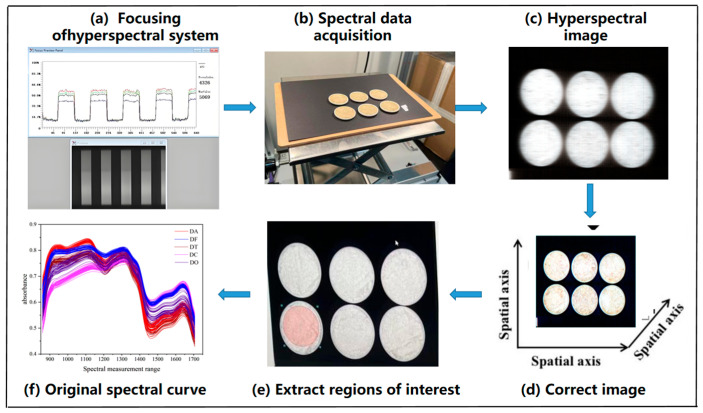
Spectrum information acquisition process.

Specifically, 2 ± 0.05 g of each sample was placed into a 30 mL ceramic evaporation dish, which was positioned flat on the moving platform and scanned at a speed of 25 mm/s. Data collection and reflectance calibration were conducted using SpecView software (Spectra VIEW 2018). Each image captured six samples simultaneously, and three replicate scans were performed for each group. The average of the three hyperspectral images was used for reflectance calibration analysis. The acquired hyperspectral images were processed using ENVI 5.6 software. This included image cropping, background masking, and spectral extraction of regions of interest (ROI). For ROI extraction, the Dendrobium powder area in each image was manually selected and cropped as the region of interest. The detailed procedure for spectral information extraction is illustrated in Figure 3.

Spectral reflectance values for all pixels within the region of interest (ROI) were extracted using ENVI 5.31 software, and the average reflectance spectrum was calculated and used as the spectral data for each sample.

### 2.4. Data Processing and Analysis

#### 2.4.1. Sample Partitioning

Common methods for sample partitioning include the Random Split (RS) method and the Kennard-Stone (K-S) algorithm. The K-S method divides samples based on their Euclidean distances in the spectral space. Samples with the greatest spectral distances are assigned to the calibration set, while the remaining samples form the prediction set. In this study, the K-S algorithm was initially applied to partition the Dendrobium samples, but it resulted in an imbalanced distribution between calibration and prediction sets across species, leading to poor model performance. This may be due to the fact that while the K-S method emphasizes spatial representativeness, it often neglects class balance, causing some categories to be significantly under- or over-represented [24]. In contrast, the RS method is simple to implement, computationally efficient, and well-suited for repeated partitioning and cross-validation. The RS method uses stratified random sampling to maintain proportional representation of each class in both the calibration and prediction sets. It does not rely on Euclidean distance, thereby reducing the risk of model performance degradation due to uneven sample distribution. Therefore, this study used the RS method to divide the dendrobium samples into calibration set, prediction set, and independent validation set in a ratio of 2:1:1 to ensure distribution balance and reliable modeling performance.

#### 2.4.2. Spectral Preprocessing

Raw spectral data not only contain information inherent to the sample but also include noise and irrelevant signals. Various preprocessing methods are employed to eliminate these interferences, thereby improving model stability and accuracy. Savitzky–Golay (SG) smoothing enhances spectral smoothness by applying a polynomial least squares fit within a moving window, effectively reducing noise while preserving spectral features. Baseline correction is a commonly used signal processing technique, especially effective for experimental data with high noise levels. It helps eliminate background signals and baseline drift. Its primary advantage lies in accurately isolating true signal variations from raw data, thereby minimizing the impact of baseline fluctuations on analytical results. In addition, the Baseline preprocessing method offers strong flexibility, allowing the use of different baseline correction strategies tailored to specific signal types. This enhances the reliability of data and the precision of subsequent analysis. As a result, baseline correction is widely applied in fields such as biomedicine, chemical analysis, and spectroscopy, serving as a key approach for improving data quality and ensuring result reproducibility. Normalization (NOR) adjusts data to a comparable scale or distribution, which helps eliminate differences in dimensional units among features. This ensures balanced weighting during analysis and improves model performance. Derivative-SGolay combines the Savitzky–Golay filter (SGolay) with derivative computation. It uses local polynomial fitting to perform both signal smoothing and derivative estimation. The SGolay filter effectively removes noise while preserving the overall trend of the signal through localized smoothing, whereas Derivative-SGolay enables direct computation of first- or higher-order derivatives by tuning filter parameters. This method is widely used in data analysis, smoothing and derivative calculation of physical experimental data, and spectral analysis for both denoising and derivative estimation. In this way, the Derivative-SGolay filter can retain essential signal features while minimizing the influence of noise on derivative estimation. In this study, the raw spectral data were preprocessed using SG, Baseline, NOR, and Derivative-SGolay methods.

#### 2.4.3. Characteristic Wavelength Selection Methods

In this study, two feature wavelength selection methods were employed: Competitive Adaptive Reweighted Sampling (CARS) and the Successive Projections Algorithm (SPA).

The CARS method is a feature selection technique inspired by the evolutionary principle of “survival of the fittest”. Its core principle is to iteratively apply adaptive reweighted sampling to select wavelength variables with large absolute regression coefficients in a partial least squares (PLS) model, while eliminating those with small weights. Cross-validation is then used to determine the optimal subset of wavelengths. The Successive Projections Algorithm (SPA) is an efficient feature selection method widely used in spectral analysis and dimensionality reduction in high-dimensional data. The core idea of SPA is to iteratively select new features that are minimally correlated with those already chosen, thereby reducing redundancy and enhancing model robustness. SPA is computationally efficient and capable of identifying key wavelengths while minimizing collinearity, which improves the generalizability and interpretability of the resulting model.

### 2.5. Classification Modeling and Evaluation Methods for Dendrobium Samples

#### 2.5.1. Modeling Method

Random Forest (RF) algorithm is a machine learning method based on ensemble learning, which has the advantages of effectively processing high-dimensional data, avoiding overfitting, high classification accuracy, and good robustness. Random forest constructs multiple decision trees and integrates their results, using “majority voting” or “averaging” strategies for final prediction. This method can provide good prediction performance when facing complex and non-linear data, and has strong fault tolerance for feature selection and handling missing values. During the training process of each tree, a portion of features and samples is randomly selected to avoid the problem of correlation between features and improve the generalization ability of the model. Especially in hyperspectral imaging technology, the random forest algorithm can effectively process high-dimensional features in imaging data, automatically extract key features, and provide accurate classification results. In addition, in the identification of traditional Chinese medicinal materials, with the wide variety and high similarity in morphology of Chinese medicinal materials, traditional identification methods often face problems of sample diversity and large errors [31]. After obtaining the spectral information of traditional Chinese medicine through hyperspectral imaging technology, random forest can classify and identify these spectral data, and use its advantages in high-dimensional data to accurately distinguish different types of traditional Chinese medicine. Through feature selection and classification of hyperspectral images, the random forest algorithm not only improves discrimination accuracy, but also effectively solves the problems of difficult sample processing and low recognition rate that may be encountered in traditional methods.

Support Vector Machine (SVM) is a supervised learning algorithm used for classification and regression analysis. Its core principle is to identify an optimal hyperplane that effectively separates different classes of samples while maximizing the margin—the distance between the hyperplane and the closest data points from each class. These closest data points are known as support vectors, which are critical for defining the position and orientation of the hyperplane. SVM performs particularly well when handling high-dimensional or non-linear data by employing kernel functions to project the input data into a higher-dimensional space, enabling non-linear separation. It offers strong generalization capabilities and robustness, making it well-suited for high-dimensional datasets with limited sample sizes.

As a supervised machine learning classification algorithm, Support Vector Machine (SVM) demonstrates notable advantages over other classification methods, particularly in tasks involving high-dimensional, non-linear, and small-sample data, such as the detection and identification of *Dendrobium officinale* and other traditional Chinese medicinal materials. Compared with traditional classifiers such as decision trees, k-nearest neighbors (KNN), and logistic regression, SVM is grounded in statistical learning theory. By constructing a maximum-margin hyperplane, it enhances model generalization and effectively reduces the risk of overfitting [32,33]. Moreover, SVM offers powerful non-linear processing capabilities. By employing kernel functions—such as radial basis function (RBF), sigmoid, or polynomial kernels—it can project non-linearly separable data from a low-dimensional space into a higher-dimensional feature space where linear separation becomes possible, enabling the effective handling of complex non-linear classification problems. In practical applications, SVM exhibits strong robustness when the number of features greatly exceeds the number of samples. It is relatively insensitive to redundant information, noise, and outliers, which contributes to enhanced model stability and accuracy. Furthermore, the SVM model has a simple structure and requires relatively few hyperparameters—primarily the penalty parameter (C) and kernel parameters. Compared with deep learning models such as neural networks, SVM is easier to implement and interpret, with lower computational cost. In the detection of *Dendrobium officinale*, SVM is often integrated with near-infrared hyperspectral imaging (NIR-HSI) to classify high-dimensional spectral information from images. Applications include variety and origin identification, detection of adulterated samples, and automated quality grading. SVM is especially well-suited to situations where sample sizes are limited but feature dimensions are high, offering a more practical solution than data-intensive models like neural networks.

#### 2.5.2. Model Evaluation

In this study, the performance and robustness of the classification models were evaluated using four metrics: recall, precision, F1 score, and accuracy. The corresponding calculation formulas are provided in Equations (1)–(4).

(1) $$\mathrm{Recall}=\frac{TP}{TP+FN}\times 100\%$$

(2) $$\mathrm{Precision}=\frac{TP}{TP+FP}\times 100\%$$

(3) $$F1\cdot S\mathrm{core}=2\times \frac{\mathrm{Precision}\times \mathrm{Recall}}{\mathrm{Precision}+\mathrm{Recall}}\times 100\%$$

(4) $$\mathrm{Accuracy}=\frac{TP+TN}{TP+TN+FP+FN}\times 100\%$$

where *TP* (True Positive) refers to the number of positive samples correctly predicted as positive; *FN* (False Negative) is the number of positive samples incorrectly predicted as negative; *FP* (False Positive) is the number of negative samples incorrectly predicted as positive; and *TN* (True Negative) is the number of negative samples correctly predicted as negative.

## 3. Results and Discussion

### 3.1. Spectral Analysis

**Figure 4:**
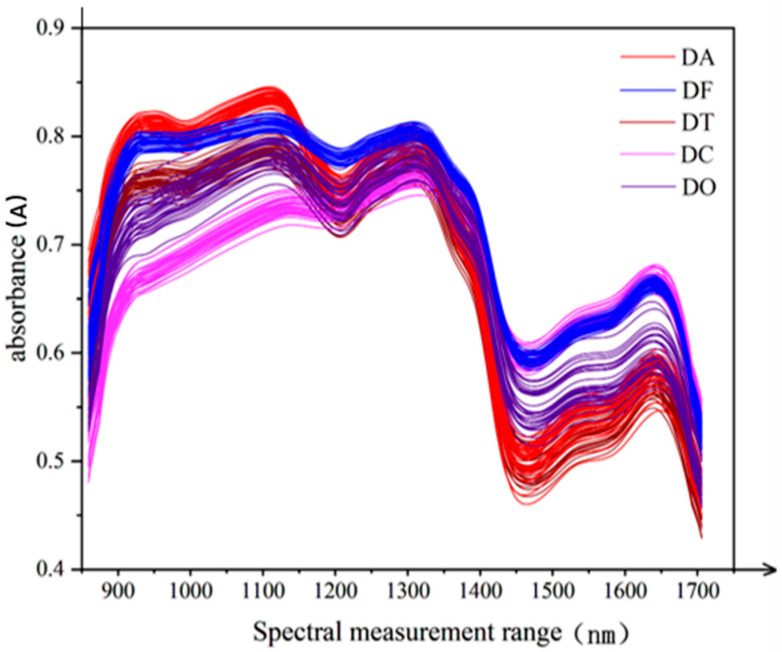
Botanical structure diagram of *Dendrobium officinale*.

Figure 4 presents the raw spectral curves of the five Dendrobium species. Although the overall spectral trends are similar across all species, notable differences are observed at specific absorption peaks, reflecting variations in chemical composition among the different types.

As shown in the figure, DA and DT exhibit relatively strong absorption peaks in the 920–960 nm range. In the 1120–1160 nm range, all species except DC show strong absorption. Furthermore, strong absorption peaks are observed for all samples within the 1310–1350 nm and 1640–1670 nm intervals. Specifically, DO, DF, DT, and DA share common characteristic absorption peaks at 1160 nm, 1127 nm, and 1105 nm—wavelengths typically associated with polysaccharides such as starch and cellulose. These species display highly similar peak positions, relative intensities, and peak shapes, suggesting that their stems are rich in structurally similar polysaccharides. In contrast, DC lacks these key absorption peaks, indicating a lower polysaccharide (starch, cellulose) content in its stem. Instead, DC appears to contain a higher proportion of monosaccharides and disaccharides. However, in certain spectral regions, the average reflectance curves of two or more species tend to overlap, making it difficult to distinguish all five Dendrobium species based solely on visual inspection of the spectral curves [8,9].

### 3.2. Classification Model Analysis Based on Full Wavelengths

To improve the quality of spectral data and evaluate the impact of various preprocessing techniques on the classification performance of different Dendrobium species, this study developed support vector machine (SVM) models and random forest algorithm (RF) for spectral data preprocessed by different methods. Then, the performance of each model was compared and analyzed.

As shown in Table 2, the SVM classification results based on raw and preprocessed spectral data varied across the calibration and prediction sets. For the calibration set, the raw spectral data yielded relatively good classification performance. For instance, DA achieved a recall of 95%, an F1 score of 95, and an accuracy of 95%, while DC reached 100% in both recall and accuracy. The classification results for the other species were also relatively satisfactory. Specifically, DF and DT achieved recall rates of 100% and 95%, and F1 scores of 90.91% and 79.17%, respectively, indicating strong performance. However, performance declined in the prediction set, particularly for DO, where the recall dropped to 30% and the F1 score to 46.15%, indicating poor generalization of the raw spectral data for this species. In comparison, spectral data subjected to smoothing preprocessing performed similarly to the raw data on the calibration set. Both DA and DC achieved 100% in recall, F1 score, and accuracy. Although DO still exhibited lower recall and F1 scores (50% and 66.67%, respectively), this represented an improvement over the raw spectral data. On the prediction set, smoothing preprocessing continued to yield good classification results for DA and DC, with DA achieving a recall of 100% and an F1 score of 95.24%. DC and DO also showed stable performance, with accuracy reaching 100%. The research results indicate that the SVM model struggles to differentiate between DT and DA samples, even after various pretreatments, suggesting spectral similarity between the two. This may stem from their close genetic relationship, which increases the likelihood of classification errors. Additionally, sample quality variability and the influence of environmental factors during collection also contribute to misclassification.

As shown in Table 3, the RF classification results based on raw and preprocessed spectral data differ between the calibration and prediction sets. For the calibration set, the raw spectral data produced relatively good classification performance. Overall, the prediction performance of the model dataset constructed based on RF algorithm is slightly worse than that of the model dataset constructed based on SVM algorithm. Among them, the model preprocessed by Normalize has the best performance, with an accuracy of 87% for the calibration set and 80% for the prediction set.

Normalization preprocessing showed strong performance on the calibration set. For DC, all three metrics—recall, F1 score, and accuracy—reached 100% in SVM classification model. DT was also identified accurately, with a recall of 100%, an F1 score of 97.56%, and an accuracy of 95.24%. However, in the prediction set, although the model achieved satisfactory classification performance for DF and other species, the recall for DA dropped to 40% with an F1 score of 57.14%, indicating that normalization may have limited generalization ability for certain species. The Savitzky–Golay smoothing method exhibited variable performance on the calibration set. For DA, the model achieved a recall of 95%, an F1 score of 97.44%, and 100% accuracy. However, for DT, the recall was only 65% with an F1 score of 78.79%, while DO achieved 100% recall but only 83.33% accuracy. On the prediction set, SGolay performed well for DA and DF, achieving 100% recall and F1 score for DA. However, for DT, the model’s recall dropped to 50%, with accuracies of 66.67% and 83.33%, indicating relatively weak generalization capability. The Baseline preprocessing method showed relatively stable performance on the calibration set. For DA, the model achieved a recall of 70%, an F1 score of 80%, and an accuracy of 93.33%. For DC, all evaluation metrics reached 100%. However, in the prediction set, the F1 score for DA decreased to 82.35%, the accuracy for DT was 71.43%, and the recall for DO was 70% with an F1 score of 82.35%, suggesting that the baseline preprocessing method had limited generalization capability.

**Figure 5:**
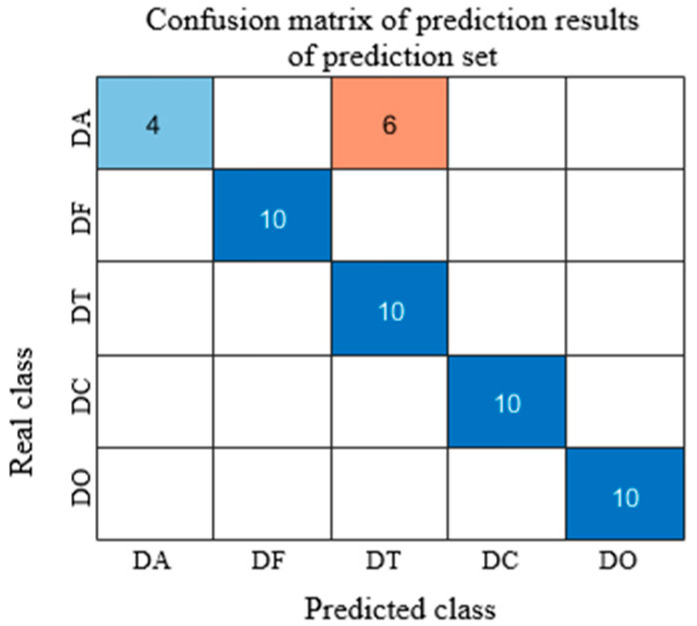
Prediction results of test set after normalization pretreatment. (The dark/light blue square represents correctly predicted samples, and the brown square represents incorrectly predicted samples).

As shown in Figure 5, after normalization preprocessing, the prediction accuracy on the test set reached 88%. However, the classification performance for DA was relatively poor, with six DA samples misclassified as DT.

Overall, the Normalize preprocessed data produced the best performance in both the calibration and prediction sets based on SVM and RF models. The Normalize-SVM model achieved the best overall performance, which may be attributed to the fact that normalization does not rely on mean or standard deviation and is more sensitive to outliers. As a result, it often performs better when the data distribution is relatively uniform and free of significant anomalies [34].

### 3.3. Classification Model Analysis Based on Feature Wavelength Selection

To further enhance the performance of the classification models, this study applied two feature wavelength selection methods—Competitive Adaptive Reweighted Sampling (CARS) and Successive Projections Algorithm (SPA)—to the spectral data preprocessed using normalization (NOR). The primary objective of feature wavelength selection is to identify spectral bands with high discriminative power, thereby reducing redundancy and noise interference, and ultimately improving the accuracy and robustness of the classification models.

#### 3.3.1. Modeling Results Based on CARS Feature Wavelength Selection

**Figure 6:**
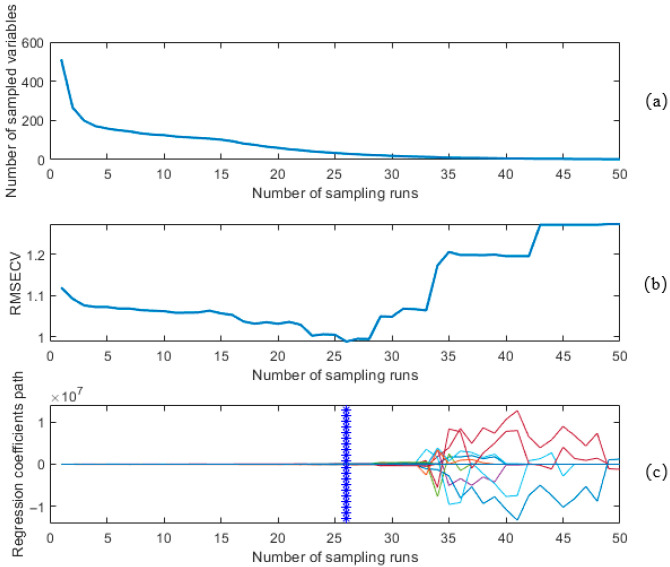
Results of CARS characteristic wavelength selection: (**a**) trends in the number of sampled features; (**b**) trends in cross-validated error; (**c**) trends in regression coefficients for wavelength variables. (The colored curves show the distribution of regression coefficients for each wavelength at different iterations. Initially, most wavelengths have non-zero coefficients (darker/cooler colors). As iterations progress (color transition), more coefficients approach zero, leaving only the key wavelengths that contribute most to the model, reflecting a gradual optimization process.).

Figure 6 shows the results of feature wavelength selection using the CARS algorithm. As shown in Figure 6a, the number of selected feature wavelengths gradually decreases with an increasing number of sampling iterations. The decline slows over time, reflecting a transition from coarse to fine wavelength selection. Figure 6b illustrates the variation in cross-validation error rate during the wavelength selection process. It can be observed that the error rate initially decreases gradually and then increases as the selection progresses. As redundant wavelengths are eliminated, the RMSE of cross-validation (RMSECV) gradually decreases and reaches its minimum at the 26th sampling iteration. After that point, the RMSECV begins to rise, possibly due to the inadvertent elimination of critical wavelengths [35]. Figure 6c illustrates the trend of regression coefficients for wavelength variables. The asterisk “\*” marks the 26th Monte Carlo (MC) sampling, where the cross-validation error rate reaches its minimum. After this point, the regression coefficients increase rapidly, even surpassing 100%, resulting in a significant decline in model performance.

After feature selection using the CARS algorithm, a total of 30 characteristic wavelength variables were retained: 862.44 nm, 867.35 nm, 880.44 nm, 890.26 nm, 926.29 nm, 927.93 nm, 929.57 nm, 954.16 nm, 955.80 nm, 957.44 nm, 959.08 nm, 960.72 nm, 962.36 nm, 965.64 nm, 1052.72 nm, 1056.01 nm, 1059.30 nm, 1074.12 nm, 1361.84 nm, 1363.50 nm, 1410.04 nm, 1413.37 nm, 1454.98 nm, 1456.65 nm, 1459.98 nm, 1583.48 nm, 1586.82 nm, 1590.17 nm, 1591.84 nm, and 1668.86 nm—accounting for 5.86% of the total spectral bands.

**Figure 7:**
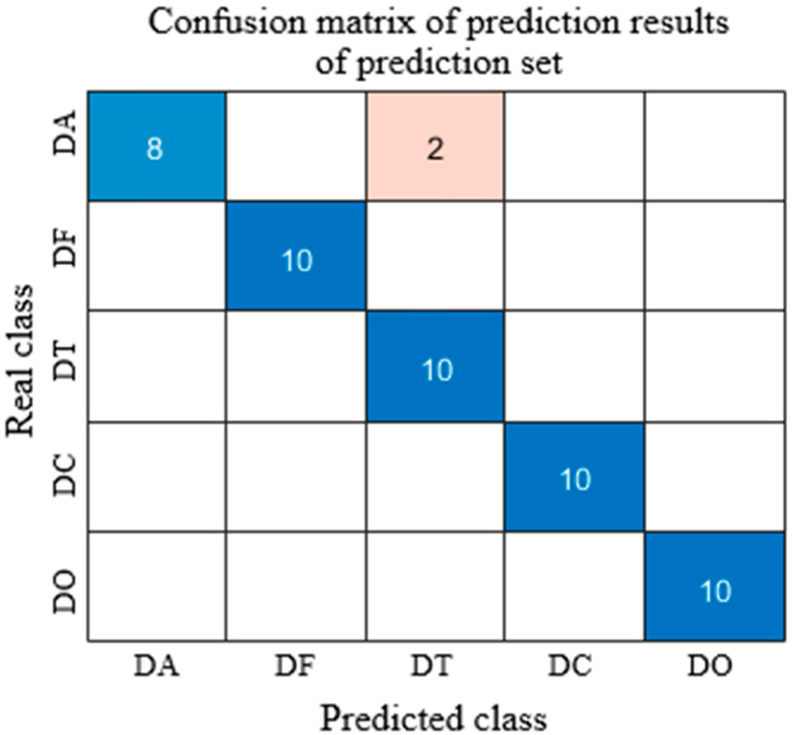
CARS-Normalize-SVM Model Prediction Set Results. (The dark/light blue square represents correctly predicted samples, and the brown square represents incorrectly predicted samples).

As shown in Figure 7, the confusion matrix of the CARS-Normalize-SVM model provides an intuitive visualization of the classification performance for each sample category. In the matrix, diagonal elements represent the number of correctly classified samples, while off-diagonal elements indicate misclassifications. The results show that the model made some errors in distinguishing the DA class, with two samples misclassified as DT, suggesting feature overlap between these two categories. To improve classification performance, future work could focus on enhancing feature extraction between these two classes or increasing sample size to optimize model training. Overall, the confusion matrix confirms the strong classification performance and robustness of the SVM model in the *Dendrobium officinale* classification task.

As shown in Table 4, the overall model accuracy improved after applying CARS for feature wavelength selection. In the calibration set, most classes achieved recall, F1 score, and accuracy values above 95%, indicating strong model fitting performance. Specifically, DF, DC, and DO all reached 100% in classification metrics within the calibration set. However, the model exhibited some degree of fluctuation in the prediction set. For example, DA achieved 100% recall, an F1 score of 95.24%, and 90.91% accuracy in the prediction set, indicating satisfactory recognition performance. In contrast, DT showed relatively poor performance, with a recall of 90%, an F1 score of only 75%, and a drop in accuracy to 64.29%, reflecting insufficient generalization capability. The case of DO was even more extreme: while the accuracy reached 100%, its recall was only 30% and the F1 score was just 46.15%, suggesting that the CARS-Normalize-SVM model significantly underperformed in identifying DO samples. The results of the independent validation set show that the model accuracy reaches 98%, with all predictive indicators of the DC sample reaching 100%. However, the recall rate and F1 score of the DO sample are relatively low, only 40% and 57.14%, respectively.

#### 3.3.2. Modeling Results Based on SPA Feature Wavelength Selection

**Figure 8:**
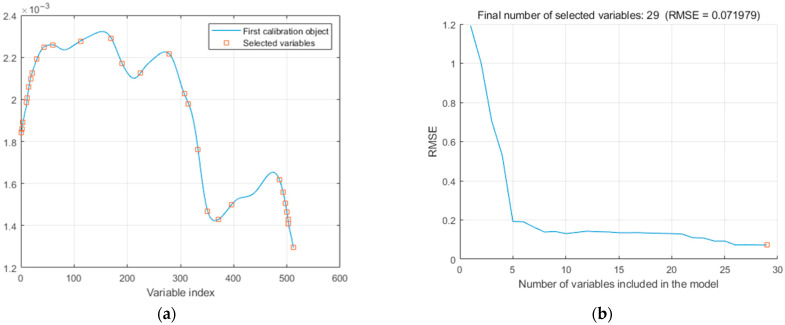
Results of SPA characteristic wavelength selection: (**a**) characteristic band distribution; (**b**) RMSE variation under different variable numbers.

After the original spectral data were preprocessed using normalization, the Successive Projections Algorithm (SPA) was applied to further simplify the model and reduce redundancy, as shown in Figure 8.

As the number of variables included in the model increased, the root mean square error (RMSE) exhibited a general downward trend. The RMSE was relatively low when 26 variables were included and reached its minimum at 29 variables, after which it tended to stabilize. In this study, a total of 29 characteristic wavelengths were selected using the SPA method: 859.17 nm, 860.81 nm, 862.44 nm, 875.53 nm, 877.17 nm, 882.08 nm, 886.99 nm, 891.90 nm, 906.63 nm, 927.93 nm, 955.80 nm, 1041.21 nm, 1136.74 nm, 1169.74 nm, 1227.58 nm, 1317.03 nm, 1363.50 nm, 1376.79 nm, 1406.71 nm, 1436.66 nm, 1469.97 nm, 1511.66 nm, 1662.16 nm, 1673.89 nm, 1680.60 nm, 1685.63 nm, 1688.98 nm, 1690.66 nm, and 1705.76 nm. These wavelengths represent approximately 5.66% of the total spectral bands. They are likely located in regions most closely associated with the chemical components of Dendrobium, particularly mannose (MN) and Dendrobium polysaccharides (DPs). By applying SPA for feature selection, data dimensionality is effectively reduced, which helps minimize the risk of model overfitting and improves the generalization capability of the classification model [36].

**Figure 9:**
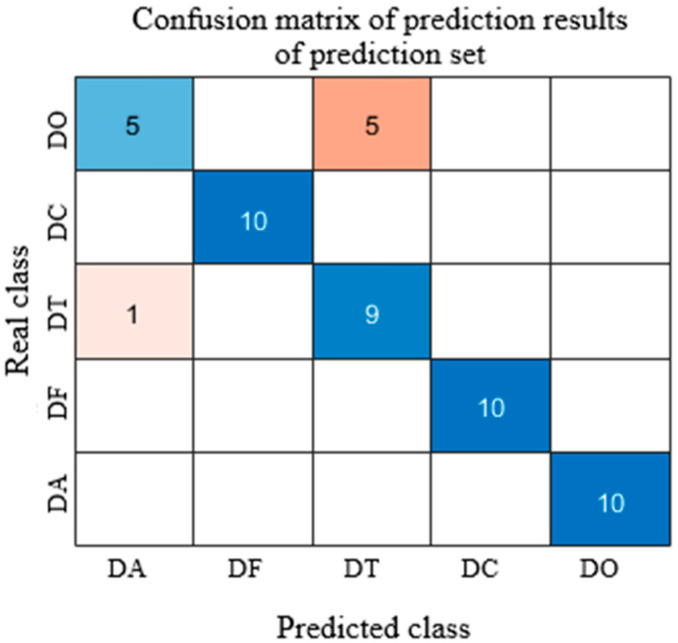
SPA-Normalize-SVM Model Prediction Set Results. (The dark/light blue squares represents correctly predicted samples. The dark/light brown squares represents incorrectly predicted samples).

As shown in Figure 9, the confusion matrix of the SPA-Normalize-SVM model clearly illustrates the classification accuracy for each sample category. The diagonal elements represent the number of correctly classified samples, while the off-diagonal elements indicate instances of misclassification. Analysis of the matrix reveals a degree of confusion between DA and DT samples: five DA samples were misclassified as DT, and one DT sample was incorrectly predicted as DA. Future improvements may include enhanced feature extraction or increased sample size for these two categories to improve class separability. Overall, the confusion matrix analysis confirms the effectiveness and robustness of the SVM model in the classification of *Dendrobium officinale*.

As shown in Table 5, the model performance exhibited certain fluctuations when feature wavelengths were selected using the SPA method. Specifically, although SPA achieved 100% accuracy for some categories such as DF and DC, its performance was less consistent for other categories, including DA, DT, and DO—particularly with regard to recall and accuracy for DA and DT. For instance, DA had a recall of 80%, an F1 score of 82.05%, and an accuracy of 84.21%, indicating relatively poor classification performance. In the case of DT, the recall and accuracy on the prediction set were only 75% and 64.29%, respectively, suggesting possible information loss during feature selection for certain classes when using SPA. The results of the independent validation set show that the model accuracy reaches 90%, with all predictive indicators of DC, DC, and DO samples reaching 100%, which is the same as the predictive indicators of the calibration set and prediction set. However, the recall rate and F1 score of DA samples are relatively low, only 60% and 70.59%, respectively.

In contrast, feature wavelength selection using the CARS method resulted in more stable and superior model performance. The DA, DF, and DC classes all achieved 100% recall, F1 score, and accuracy, and the model’s accuracy on the prediction set also approached or reached 100%. For DA in particular, the model achieved 95% recall, a 95% F1 score, and 95% accuracy in the calibration set, with an accuracy of 98% on the prediction set, demonstrating excellent classification capability.

Overall, the SVM models built using selected feature wavelengths outperformed those based on full-spectrum data, demonstrating that feature wavelength extraction not only significantly reduces data dimensionality but also improves computational efficiency. Compared to SPA, the CARS-based models showed superior classification accuracy in both calibration and prediction sets. This is likely because CARS adaptively selects informative wavelengths while eliminating redundant variables, thereby enhancing overall classifier performance. This finding differs from the conclusion reported by Shao et al. [37], who found the SPA-SVM model to be more effective. The discrepancy may be attributed to methodological differences: Shao et al. employed principal component analysis (PCA) to perform cluster analysis on Dendrobium samples prior to feature wavelength selection, which may have influenced their results differently from those of the present study.

## 4. Conclusions

This study proposed a rapid identification method for different Dendrobium species based on near-infrared hyperspectral imaging (NIR-HSI). Hyperspectral image data were collected and analyzed for five Dendrobium species: *D. officinale*, *D. aphyllum*, *D. chrysanthum*, *D. fimbriatum*, and *D. thyrsiflorum*. Combined with various data preprocessing techniques and classification models, efficient and accurate classification models were established for these species. Several spectral preprocessing methods were applied, and support vector machine (SVM) and random forest (RF) algorithms were used for classification to evaluate the impact of different preprocessing strategies on the performance of the two models. The results indicate that the SVM-based classification model can more reliably distinguish different Dendrobium species. Among the preprocessing techniques, normalization (Normalize) achieved the highest accuracy and stability, particularly for DA and DC samples, with both recall and accuracy approaching 100%. In addition, two feature wavelength selection methods—Competitive Adaptive Reweighted Sampling (CARS) and Successive Projections Algorithm (SPA)—were employed to further optimize model performance. Among them, CARS showed superior classification results after wavelength selection, effectively reducing data redundancy and improving the classification efficiency of the model. In summary, this study confirms the potential of NIR-HSI technology for rapid, non-destructive, and high-efficiency identification of Dendrobium species. Future work could focus on refining classification algorithms and feature selection strategies to further enhance recognition accuracy, providing robust technical support for Dendrobium quality control and market supervision.

## 5. Discussion

A key insight of this study is the significant impact of spectral preprocessing on model performance. Among various preprocessing methods explored, normalization has become the most effective strategy, resulting in the highest classification accuracy and stability. This result is consistent with some existing studies that demonstrate the benefits of normalization in improving the performance of machine learning models, especially when dealing with hyperspectral data that is often affected by lighting and spectral distortion. The almost perfect recall and accuracy observed for *D. aphyllum* (DA) and *D. chrysanthum* (DC) samples further confirm the robustness of the normalization technique. This study constructed classification models based on RF and SVM algorithms, and the results showed that the SVM model can more effectively handle high-dimensional data. Although current research shows promising results, there are still several areas that need further improvement. For example, expanding the dataset to include more species of Dendrobium and environmental variations can further validate the universality of our research method. Finally, future research can focus on improving the computational efficiency of feature selection and classification processes. As hyperspectral imaging generates a large amount of data, the computational burden of feature extraction and model training may become a limitation. Therefore, optimizing algorithms to efficiently process large-scale hyperspectral datasets will be a key step in making this technology easier to apply in practice.

## Figures and Tables

**Table 1:** Technical parameters of near-infrared hyperspectral imaging system.

System Components	Technical Index	Parameter Values
Imaging Spectrometer	Model	GaiaField-N17E, (Shuangli Hepu Technology Co., Ltd., Wuxi, China)
	Spectral range	870~1720 nm
	Spectral resolution	5 nm
	Spectral sampling points	3 nm
	Slit size	30 μm × 12.5 nm
	Focal length	30 mm
	Stray	<0.45%
	Luminous Efficiency	>50%
	Relative aperture	F/2.0
Imaging Lens	Type	HSIA-OLES30, (Shuangli Hepu Technology Co., Ltd., Wuxi, China)
	Transmittance	≥90%
	Field of view length	300 nm
Camera	Model	HSIA-CT, (Shuangli Hepu Technology Co., Ltd., Wuxi, China)
	Full frame pixel count (spatial Dimension x spectral dimension)	270 × 310
	Time of exposure	1~500 ms
Calibrate Whiteboard	Size	150 mm × 150 mm
tungsten-halogen lamp	Model	ModelsXC-130, (Shuangli Hepu Technology Co., Ltd., Wuxi, China)
	Power	150 W
Electric Displacement Platform	Model	PSA200-11-X, (Shuangli Hepu Technology Co., Ltd., Wuxi, China)
	Transmission speed	0~25 mm/s

**Table 2:** Results of establishing SVM models to distinguish different varieties of Dendrobium under different preprocessing methods.

Pretreatment	Type	Calibration Set				Prediction Set			
		Recall (%)	F1 Score (%)	Precision (%)	Model Accuracy (%)	Recall (%)	F1 Score (%)	Precision (%)	Model Accuracy (%)
Raw	DA	95	95	95	86	100	95.24	90.91	84
	DF	100	90.91	83.33		100	90.91	83.33	
	DT	95	79.17	67.86		90	75	64.29	
	DC	100	100	100		100	100	100	
	DO	40	57.14	100		30	46.15	100	
Smoothing	DA	95	95	95	88	100	95.24	90.91	84
	DF	100	95.24	90.91		100	90.91	83.33	
	DT	95	79.17	67.86		90	75	64.29	
	DC	100	100	100		100	100	100	
	DO	50	66.67	100		30	46.15	100	
**Normalize**	**DA**	**95**	**97.44**	**100**	**97**	**40**	**57.14**	**100**	**88**
	**DF**	**100**	**95.24**	**90.91**		**100**	**100**	**100**	
	**DT**	**100**	**97.56**	**95.24**		**100**	**76.92**	**62.5**	
	**DC**	**100**	**100**	**100**		**100**	**100**	**100**	
	**DO**	**90**	**94.74**	**100**		**100**	**100**	**100**	
SGolay	DA	95	97.44	100	89	100	100	100	86
	DF	85	91.89	100		80	88.89	100	
	DT	65	78.79	100		50	66.67	100	
	DC	100	93.02	86.96		100	90.91	83.33	
	DO	100	83.33	71.43		100	80	66.67	
Baseline	DA	70	80	93.33	87	70	82.35	100	88
	DF	95	90.48	86.36		100	90.91	83.33	
	DT	90	78.27	69.23		100	83.33	71.43	
	DC	100	100	100		100	100	100	
	DO	80	86.49	94.12		70	82.35	100	

**Table 3:** Results of establishing RF model to distinguish different varieties of Dendrobium under different preprocessing methods.

Pretreatment	Type	Calibration Set				Prediction Set			
		Recall (%)	F1 Score (%)	Precision (%)	Model Accuracy (%)	Recall (%)	F1 Score (%)	Precision (%)	Model Accuracy (%)
Raw	DA	85	85	85	77	90	87.8	85.71	76
	DF	90	81.82	75		90	83.33	76.19	
	DT	85	70.83	60.71		80	69.57	59.26	
	DC	90	90	90		100	95.24	90.91	
	DO	35	51.85	87.5		30	42.86	85.71	
Smoothing	DA	85	85	85	79	90	87.8	85.71	76
	DF	90	85.71	81.82		90	83.33	76.19	
	DT	85	70.83	60.71		80	69.57	59.26	
	DC	90	90	90		100	95.24	90.91	
	DO	45	60	81.82		30	42.86	85.71	
Normalize	DA	85	87.18	89.47	87	40	52.17	85.71	80
	DF	90	85.71	81.82		90	91.84	90	
	DT	90	87.8	85.71		90	70.59	57.14	
	DC	90	90	90		100	95.24	90.91	
	DO	80	85.11	90.91		90	91.84	90	
SGolay	DA	85	87.18	89.47	80	90	91.84	90	78
	DF	75	82.76	92.31		70	81.82	90	
	DT	55	70.97	91.67		40	60.87	87.50	
	DC	90	83.72	78.26		90	83.33	76.19	
	DO	90	75	64.29		90	73.47	60	
Baseline	DA	65	72.22	81.25	78	60	75	90	80
	DF	85	81.4	77.78		90	83.33	76.19	
	DT	80	70.59	63.16		90	76.19	64	
	DC	90	90	90		100	95.24	90.91	
	DO	70	77.78	87.50		60	75	90	

**Table 4:** SVM modeling results based on feature wavelength selection of preprocessed data using CARS.

Method	Type	Calibration Set				Prediction Set				Independent Validation Set			
		Recall (%)	F1 Score (%)	Precision (%)	Model Accuracy (%)	Recall (%)	F1 Score (%)	Precision (%)	Model Accuracy (%)	Recall (%)	F1 Score (%)	Precision (%)	Model Accuracy (%)
CARS	DA	95	95	95	98	100	95.24	90.91	96	100	97.09	94.34	98
	DF	100	100	100		100	90.91	83.33		100	92.31	85.71	
	DT	95	95	95		90	75	64.29		90	76.60	66.67	
	DC	100	100	100		100	100	100		100	100.00	100.00	
	DO	100	100	100		30	46.15	100		40	57.14	100.00	

**Table 5:** SVM modeling results based on feature wavelength selection of preprocessed data using SPA.

Method	Type	Calibration Set				Prediction Set				Independent Validation Set			
		Recall (%)	F1 Score (%)	Precision (%)	Model Accuracy (%)	Recall (%)	F1 Score (%)	Precision (%)	Model Accuracy (%)	Recall (%)	F1 Score (%)	Precision (%)	Model Accuracy (%)
SPA	DA	80	82.05	84.21	93	50	62.50	83.33	88	60	70.59	85.71	90
	DF	100	100	100		100	100	100		100	100	100	
	DT	85	82.93	80.95		90	75	64.29		90	78.26	69.23	
	DC	100	100	100		100	100	100		100	100	100	
	DO	100	100	100		100	100	100		100	100	100	

## Data Availability

Data available upon request.
